# Foot segments mobility and plantar pressure in the normal foot

**DOI:** 10.1186/1757-1146-7-S1-A11

**Published:** 2014-04-08

**Authors:** Paolo Caravaggi, Claudia Giacomozzi, Alberto Leardini

**Affiliations:** 1 Movement Analysis Laboratory, Istituto Ortopedico Rizzoli, Bologna, 40136, Italy; 2 Department of Technology and Health, Istituto Superiore di Sanità, Roma, 00161, Italy

## Background

The foot is generally regarded as a flexible structure which can adjust its flexibility in response to variable dynamic conditions in different phases within different motor tasks. In gait, both kinematics and baropodometry have shown to be affected by functional and structural factors [1]. In fact pressure distribution can be seen as the effectiveness of the musculoskeletal system in absorbing the ground reaction forces via the foot and its joints. Excessive foot pressure may develop into calluses, which become sites of peak pressure and pain. The relationship between foot joints mobility and plantar pressure has not been thoroughly investigated. Aim of this study was to combine a multi-segment kinematics model [2] and baropodometric analysis based on anatomical masking [3], to investigate correlations between intersegmental kinematics and regional baropodometric parameters in the normal foot.

## Materials and methods

**Figure 1:**
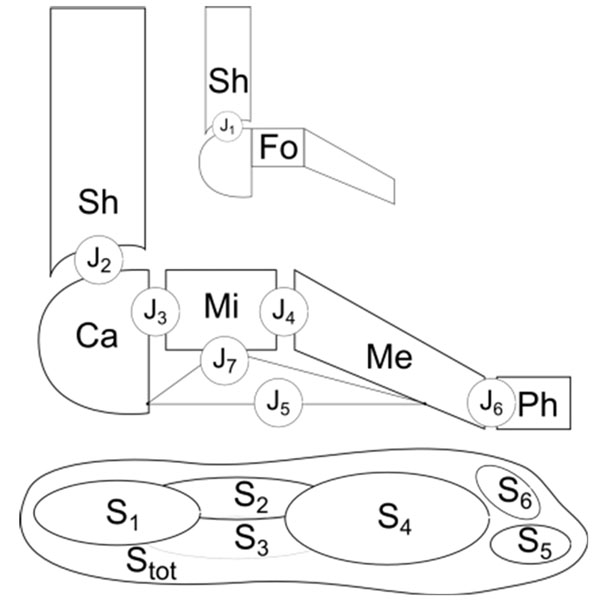
Schematic drawing of the seven foot joints (J1-J6 and the medial longitudinal arch angle J7), according to [2], and of the footprint subareas (S1-S6) as follows: S1, rearfoot; S2, lateral midfoot; S3, medial midfoot; S4, forefoot; S5, hallux; S6, 2-5 toes. Stot is the total footprint area.

Ten able-bodied subjects (26.8 ± 6.9 years; 67.5 ± 12.6 Kg) volunteered in the study. An eight-camera motion system (Vicon, UK) was used to track foot segments during the stance phase of level walking, according to an established protocol (Figure 1, top) [2]. Simultaneously, a pressure plate (Novel, Gmbh) recorded foot plantar pressure over three repetitions. An anatomical-based selection of areas of interest was employed to divide the pressure footprints in seven subareas (Figure 1, bottom) [3]. Maximum of mean and peak pressure, of vertical force, contact-area and -time, and pressure- / force-time integrals, were determined for each subarea. The relationship between range of motion (ROM) of each foot joint and baropodometric parameters in each subarea was investigated using Pearson’s and Spearman’s coefficients.

## Results

**Figure 2:**
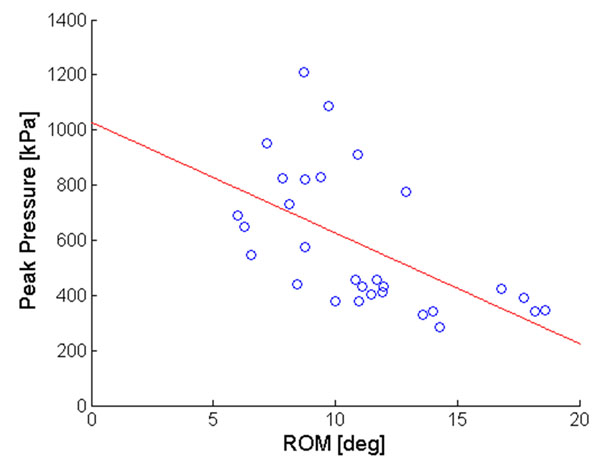
Scatter plot of the relationship between peak pressure (kPa) at the forefoot (S4 in fig. 1) and sagittal-plane ROM (deg) at the tarso-metatarsal joint (J4 in fig.1). The linear regression line is superimposed to the data points.

Most of the statistically significant correlations (p<0.05) between foot joints ROM and baropodometric parameters were moderate (|R| =0.36 – 0.67). In general, mean and peak pressure at rearfoot and forefoot were negatively correlated with the amount of motion at the ankle and tarso-metatarsal joints (Figure 2). In contrast, pressure at the hallux and midfoot were positively correlated with the ROM of the joints across the midfoot. Strong correlation was found between ROM of the medial longitudinal arch angle (J7) and pressure-time-integral at the forefoot (Spearman Rho = - 0.93, p<0.05).

## Conclusions

According to the sample of normal feet analyzed in this study, those feet presenting smaller joint mobility are associated with larger pressure at the rear- and forefoot. A trend for decreased pressure at the midfoot and toes was also detected in feet with a stiffer medial longitudinal arch.
